# Beyond health system contact: measuring and validating quality of childbirth care indicators in primary level facilities of northern Ethiopia

**DOI:** 10.1186/s12978-020-00923-w

**Published:** 2020-05-24

**Authors:** Haftom Gebrehiwot Weldearegay, Araya Abrha Medhanyie, Hagos Godefay, Alemayehu Bayray Kahsay

**Affiliations:** 1 College of Health Sciences, Mekelle University, Mekelle, Ethiopia; 2 Tigray Region Health Bureau, Mekelle, Tigray Ethiopia

**Keywords:** Quality, Validating indicators, Content of child birth and primary level care

## Abstract

**Background:**

Measurement of quality of health care has been largely overlooked and continues to be a major health system bottleneck in monitoring performance and quality to evaluate progress against defined targets for better decision making. Hence, metrics of maternity care are needed to advance from health service contact alone to content of care. We assessed the accuracy of indicators that describe the quality of basic care for childbirth functions both at the individual level as well as at the population level in Northern Ethiopia.

**Methods:**

A validation study was conducted by comparing women’s self-reported coverage of maternal and newborn health interventions during intra-partum and immediate postpartum care received in primary level care facilities of Northern Ethiopia against a gold standard of direct observation by a trained third party (*n* = 478). Sensitivity, specificity and individual-level reporting accuracy via the area under the receiver operating curve (AUC) and inflation factor (IF) to estimate population-level accuracy for each indicator was applied for validity analysis.

**Findings:**

455(97.5%) of women completed the survey describing health interventions. Thirty-two (43.2%) of the 93-basic quality child birth care indicators that were assessed could be accurately measure at the facility and population level (AUC > 0.60 and 0.75 < IF< 1.25). Few of the valid indicators were: whether women and their companion were greeted respectfully, whether an HIV test was offered, and whether severe bleeding (hemorrhage) was experienced by the woman. An additional 21(28.4%) indicators accurately measure at the facility or individual level, but the indicators under or over estimate at population level. Thirteen other indicators could accurately measure at population level. Eight (8.6%) indicators didn’t meet either of the validity criteria.

**Conclusion:**

Women were able to accurately report on several indicators of quality for basic child birth care. For those few indicators that required a technical understanding tended to have higher don’t know response from the women. Therefore, valid indicators should be included as a potential measurement of quality for the childbirth care process to ensure that essential interventions are delivered.

## Plain text summary

**What is already known on this topic?** As facility deliveries increase and the global community pays greater attention to service quality, observation as a clinical quality assessment tool may be a valuable measure. However, the existing observation-based measures are lengthy, introduce the possibility for measurement error and are difficult to administer.

**What does this study add?** Our finding revealed that women were able to accurately report on several (*n* = 32) of the 93 basic quality child birth care indicators across phases of labor and delivery. A few of them were: whether women and their companion were greeted respectfully, whether an HIV test was offered, and whether severe bleeding (hemorrhage) was experienced by the woman. An additional 21 indicators met the facility-level accuracy, 13 met the population level accuracy and 8 indicators did not meet either of the criteria.

Indicators that met both validity criteria (AUC and IF) could be appropriate for the measurement of quality for care at the facility and population level.

Indicators that do not meet criteria for the AUC but do meet the IF criterion may be suitable to measure intervention coverage at the population level as false positive and negative reporting balance each other at the aggregate level.

Moreover, indicators that do met AUC criteria, but not IF criteria may be useful for facility level measurement or useful for individual level classification but may be over reported at the population level. However, indicators that met neither of the criteria are invalid for measurement purposes, but should be used in accordance with the rationale for their distinctive use.

**What are the implications for practice and further research?**

These accurate indicators are very important for other researchers as a potential measurement of quality of routine child birth care signal functions in the Ethiopian setting. Despite this, there have been few efforts to develop standardized metrics of quality for both mothers and newborns throughout the continuum of maternity.

Therefore, a further nationwide survey should be conducted to facilitate movement toward a collection of fewer but better metrics of quality (content) of care indicators across the phases of labor and delivery services.

## Background

The time encompassing labor, delivery and the first 24 h after birth is the highest risk period for maternal and neonatal health. Adhering to the standards of maternity care services is essential to ensure quality services are delivered [1, 2]. In low and middle-income country settings, where the vast majority of maternal and newborn deaths occur, data on the coverage estimates of routine facility childbirth interventions often rely on the contact of skilled birth attendant for monitoring purposes [3, 4]. But the presence of a skilled birth attendant does not guarantee the actual content of care [5, 6]. Research findings indicate that measuring interventions that a woman actually receives is more informative than measuring contact with care providers, provided the women can accurately report this information [4].

Measurement of the quality of processes for basic care of childbirth interventions is complex and requires attention to empirical validation of the indicators to strengthen the quality of measurements [7]. Studies have documented the poor quality and limited sensitivity of obstetric facility records and databases for assessing the performance of care processes in both low and high-resource settings [8].

Absence of health monitoring systems that can provide accurate data on population coverage or demographic health survey programs collect inadequate information of content of care received during facility childbirth. Additionally, several researchers have highlighted discrepancies between contact with care providers and receiving quality care [9, 10]. This research notes that a number of composite measures or checklists have been developed through expert opinion, but few have been validated. They suggest that empirical validation is important in strengthening quality measures [11]. While direct observations of clinical care is considered the gold standard for measuring the quality of care, the existing observation-based measurement of childbirth process of care are lengthy, at times including hundreds of indicators [12]. This complexity introduces the possibility of measurement error, difficulty of administration, costliness, and lack of feasibility for routine use in most resource poor settings [13, 14]. In addition, we were unable to identify a publication describing a tangible study on validity of quality indicators of basic childbirth care interventions in Ethiopian settings.

Therefore, it is important to identify alternate indicators that describe the actual basic content of child birth care that can be reported accurately to be included in facility or routine data collection programs and population based-surveys. The aim of this study is to determine which aspects of basic child birth process of care indicators are able to differentiate between the two sets of measures (women’s self-reports compared against third-party observations), to explore whether women can appropriately report on these indicators and provide suggestions for modifications to data collection procedures that could advance the measurement of maternity care. This investigation also provides an opportunity to apply the results of this study for tracking progress and to enhance the monitoring of effective coverage of essential and basic interventions for both mothers and newborns.

## Methods

### Study setting and population

A facility-based cross-sectional validation study was conducted among primary health care facilities of South Eastern zone of Tigray, Northern Ethiopia. The zone has four rural districts, namely Degua Tembien, Enderta, Saharti Samre and Hintalo Wajrat. In the districts, there are a total of 4 primary hospitals and 27 health centers [15]. At the time of the study, nearly a quarter of the populations (23.4%) were of reproductive age (15–49) years. According to the 2016 Ethiopian Demographic Health Survey, nationally 26% and in the Tigray region 57% of mothers delivered in a health facility [16]. Reproductive-age group mothers who received labor and delivery care in primary health care facilities were the source population.

### Indicator selection

To identify indicators to be validated, reviews and scans of published and grey literature focused on indicators of the content of care received during facility child birth; this review was conducted between April and June 2018. Indicators were identified by a key term search of maternal health, safe motherhood, quality of care, indicator, valid, skilled birth attendant, obstetric, and intra-partum care. After collecting a list of 112 indicators, a group of reproductive health experts identified 93 key dimensions or set of indicators for validity testing [Additional file 1].

The validation indicators were selected based on the frequency of use and/or potential to assess the essential elements of mothers and newborns. The final indicators were placed into one of four categories: (1) respectful maternity care; (2) content of care; (3) non-indicated obstetrics care; and (4) maternal-neonatal outcome sections.

### Sample size

Buderer’s formula is used for sample size calculation in diagnostic accuracy studies at the required absolute precision level for sensitivity and specificity [17, 18]. Considering the proportion of mothers who received essential care practices at childbirth was 35.7% from a prior study in India [19], type 1 error was set at α = 0.05, considering a sensitivity level of 80%, a precision of 6%; specificity of 60% and a 5% non-response rate were used. As a result of all these conditions, a target sample size of 478 laboring women to be observed was calculated.

### Sampling technique and participant recruitment

The South-Eastern zone of Tigray region was selected purposely. All health centers with their respective catchment primary hospitals were included. The total sample size of the delivering women was distributed over each of the health facilities proportional to their sample size considering the average number of deliveries per facility per month and all the skilled birth attendants consented to participate in the study were enrolled. Finally, a consecutive sampling technique was used in which every laboring mother meeting the criteria of inclusion (normal first stage of labor) is selected until the required sample size was achieved. Each skilled birth attendant was observed 3–5 times.

### Data collection procedure

Data collection was conducted between July 15, 2018 and October 5, 2018. Twelve pairs of midwives, health officers and nurses worked as data collection teams constituting one observer and one interviewer for each facility. Data collectors had previous research experience and were trained for four days. The team worked in two shifts (day and night). Providers were observed by a third party consisting of trained data collectors using a structured checklist. An indicator matrix or structured checklist [Additional file 2] was developed from the Ethiopian basic emergency obstetrics guidelines [20] and published literature [4, 5, 9]. The interview questionnaires were translated into the appropriate local language “Tigrigna” and underwent minor modifications to improve local understanding and clarity for participants. Moreover, for few of the technical questions special emphasis was given in how the mothers could be easily understood by their local language expressions. The method of observation was nonintrusive, where the health care providers (HCPs) did what they normally do without being interrupted or disturbed by the observer. Observations were used as the reference standard as they reflected all facets of care including all interactions between the women and the providers. Data were again collected using exit interviews with an interviewer-based questionnaire from the delivered mother at the time of facility discharge. Interviewers and observers were not the same individuals and were external to the study facilities to reduce the possible social desirability bias.

### Statistical analysis

For each participant, a unique identification code for the client exit interview and observation record was matched. Questions of basic intervention indices were coded one if the response was performed “Yes”, zero for “No” responses and all other responses were coded as “don’t know (DK)”. Unmatched cases, missing and “DK” responses by the woman, as well as indicators that had less than five counts per cell were excluded from further validation analysis because of not fulfilling the assumptions of the validity analysis criteria. We assessed two aspects of indicator validity (accuracy of the women’s reports against the observer). The first one is accuracy at the individual or facility level, calculated as the area under the curve (AUC) which is a plot of the sensitivity (i.e., true positive rate) versus 1-specificity (i.e., true negative rate).

AUC scores range 0 to 1, with an AUC of 0.5 representing a random guess and AUC of 1 representing perfect diagnostic accuracy. For the purposes of this study we used an AUC of 0.6 or greater as a priori benchmark of validity testing [9].

The second measure of validity is to estimate the prevalence of the indicator that would be obtained from a population-based survey or population level accuracy, calculated based on the sensitivity and specificity of each indicator to its true prevalence (i.e., observer report) using the following equation: Population based prevalence = true prevalence x (sensitivity + specificity − 1) + (1- specificity). Inflation factor (IF), or the ratio of the survey-based prevalence to the true prevalence, is estimated to assess the degree to which each indicator would be over or under- estimated at the population level [14]. A priori validation criteria for the IF was set at 0.75 < IF< 1.25. In order to summarize indicator validity based on reports from women’s giving birth, we considered meeting both the individual-level (AUC) and population-level (IF) criteria (0.60 < AUC and 0.75 < IF < 1.25) [12, 21, 22]. All analysis was performed using Stata Version 14 software [23].

## Results

### Sample descriptive characteristics

Overall, 478 women admitted for labor and delivery were consented to participate. Of those who consented to participate, a total of 467 women were enrolled in the study. Among those enrolled, 2.5% (*n* = 12) were lost to follow-up or discontinued their participation. Finally, a total of 455 observer reports and client exit interviews were accurately matched and analyzed.

### Socio-demographic characteristics of participants

The mean age of women was 28 years (SD = 6.38), and ranged between 17 and 45 years. Over a third of women (41%) reported no formal education. Around 95.4% (*n* = 434) of women received antenatal care provided by skilled health personnel for reasons related to pregnancy at least once during their current pregnancy [Table 1].

**Table 1:** Percent distribution of women by background characteristics in Northern Ethiopia, *N* = 455

Indicator	Frequency	Percent
**Age**		
15–19	27	5.9
20–24	114	25.1
25–29	112	24.6
30–34	109	24.0
35–39	72	15.8
40+	21	4.6
**Educational level**		
None	187	41.1
Primary	148	32.5
Secondary	104	22.9
Higher	16	3.5
**Marital status**		
Single/never married	21	4.6
Married	410	90.1
Divorced	22	4.8
Widowed	2	0.4
**Religion**		
Orthodox Christian	430	94.5
Muslim	19	4.2
Others^a^	6	1.3
**Parity (Total live births)**		
1	75	16.5
2	112	24.6
3	98	21.5
4 or more	170	37.4
**Had antenatal care visit for the current pregnancy?**		
Yes	434	95.4
No	21	4.6

^a^Catholic and Protestants

### Validation results for recognized indicators of quality childbirth care signal functions

Based on a woman’s report about her experience of care during childbirth, of the recognized quality care indicators (*n* = 93) for validity analysis, 14 had a greater than 5% DK response by women and 5 indicators did not fulfill adequate cell size (i.e., at least 5 counts per cell). The latter five indicators included the use of enema, pubic shaving, slapping the newborn, something other than breast milk given to the baby in the first hour of birth and recording the birth weight of the newborn.

Finally, 32 indicators met both validation criteria, 21 indicators met individual - level, 13 indicators met population-level and 8 indicators didn’t meet either of the criteria. About women’s responses: a high percentage of women who responded “DK” were for the indicator of Apgar score (43.5%). While minimal “DK” responses were reported for the indicator that the provider palpate the woman’s uterus 15 min following delivery of the placenta (5.93%). All the indicators of DK women’s response were lying in the content of child birth quality indicator category [Table 2].

**Table 2:** Indicators with greater than 5% “Don’t Know” of women’s response in Northern Ethiopia, (*N* = 455)

Indicator	Women’s Self-Report Prevalence (%)	Observer Prevalence (%)	Women’s Don’t Know Responses ***n*** (%)
After the delivery of your baby, did the provider measure the Apgar score within 1st and 5th minutes?	50.11	69.89	198(43.52)
Was your baby weighed and did the provider tell you the weight of your baby?	5.46	12.16	167(41.44)
While you were in the health facility for the birth of your baby, did the provider record the activities done?	50.55	84.40	185(40.66)
Did the health care provider monitor your progress of labor through Parthograph?	47.69	57.80	175(38.46)
Immediately after your placenta was expelled, did the provider examine it?	54.51	58.90	152(33.41)
Did the provider dry the cord or use of Chlorhexidine?	38.24	42.86	106(23.30)
Did the provider apply controlled cord traction?	62.64	82.20	109(23.96)
After you gave birth, did anyone give your baby an injection called “vitamin K”?	57.8	62.42	77(16.92)
After your stay in the postnatal room, were you checked by a senior staff member of the facility before you were discharged?	52.09	40.66	76(16.70)
When you were transferred to the delivery room, did you find the delivery coach/bed was clean?	82.64	65.27	55(12.09)
Did the provider take your pulse during your labor?	59.78	61.32	43(9.45)
Did the provider perform a rapid initial assessment when you arrived to health facility?	88.37	58.24	39(8.57)
After you gave birth, did anyone check your perineum for any kind of laceration?	70.77	58.90	32(7.03)
Did the provider palpate your uterus 15 min following delivery of the placenta?	77.36	74.73	27(5.93)

The subsequent findings report validated quality of care during child birth indicators in accordance to: (1) Respectful maternity care, (2) Content of care, (3) Non-indicated care practice and (4) Maternal and newborn outcomes.

### Respectful maternity care indicators

Of the 18 indicators that reflected features of respectful maternity care (see Additional file 1 for the list), seven met both acceptable validity criteria. These were: women and their companions were greeted respectfully (AUC = 0.61, 95% CI:0.56–0.66, IF: 1.24), the provider actively listened (AUC = 0.66, 95% CI: 0.61–0.70, IF: 1.17), the woman was allowed to have a companion of her choice during labor (AUC = 0.61, 95% CI:0.52–0.67), IF: 1.24), the woman was allowed to ambulate during labor (AUC = 0.66, 95% CI:0.61–0.70), IF: 1.10), the woman was encouraged to drink or eat during labor (AUC = 0.63, 95% CI:0.58–0.69), IF: 1.18), privacy was provided during clinical care (AUC = 0.64, 95% CI:0.58–0.68), IF: 1.21) and the provider spent enough time with the mother (AUC = 0.62, 95% CI:0.57–0.66), IF: 1.09).

Three respectful maternity care indicators had accuracy at the individual or facility level. These were (from highest AUC to lowest): the provider introduced him or herself to the woman (AUC = 0.68, 95% CI: 0.63–0.72), the provider encouraged the woman to assume different positions during labor (AUC = 0.63, 95% CI: 0.58–0.68) and at least once, the provider explained what will happen during labor (AUC = 0.61, 95% CI: 0.56–0.65). The prevalence for each indicator as reported by the women and the observer were incongruent for these indicators. For example, 54.29% of the women surveyed reported that the provider introduced one’s own name and role, while only 23.96% of the observers reported this with low specificity (Sp = 54.34, 95% CI: 48.92–59.67).

Four respectful maternity care indicators showed population-level accuracy: The provider responded professionally (AUC = 0.58 ± 0.05, IF: 1.08), the provider did not physically abuse the patient (AUC = 0.61 ± 0.06, IF: 1.08), the provider did not abandon patient without care (AUC = 0.52 ± 0.04, IF: 0.75), and the provider maintained good communication/collaboration (AUC = 0.59 ± 0.05, IF: 1.03). Those population level accurate indicators had high false positive rate with high sensitivity ranges from 85.86 to 97.26% and low specificity that ranges (18.82–40.96%). Four respectful maternity care indicators did not meet either of the validity criteria [Table 3]: women provided oral consent before examination (AUC = 0.58 ± 0.05, IF: 1.33), were allowed to have a companion during delivery (AUC = 0.50 ± 0.04, IF: 1.26), providers did not verbally abuse their patient (AUC = 0.51 ± 0.04, IF: 1.29) and providers treated clients equally without discrimination (AUC = 0.57 ± 0.05, IF: 1.31). These indicators did not meet either of the validity criteria.

**Table 3:** Validation Results on Respectful Maternity Care Quality Indicators in Northern Ethiopia (*N* = 455)

Indicators	Women’s reported Prev (%)	True prev (%)	Sensitivity of women’s self-report (95% CI)	Specificity of women’s self-report (95% CI)	Population survey estimate (Pr)	AUC (95% CI)	IF	Criteria Met
Women and companion were greeted respectfully	81.54	59.78	90.81(86.73–93.96)	31.69(25.03–38.97)	74.13	0.61(0.56–0.66)	1.24	Both
Provider actively listened to the women	82.20	70.33	91.56(89.60–94.11)	39.26(30.97–48.03)	82.42	0.66(0.61–0.70)	1.17	Both
Women allowed to have a companion of choice during labor	77.36	63.08	84.32(79.59–88.33)	32.14(25.16–39.77)	78.24	0.61(0.52–0.67)	1.24	Both
Women were encouraged to ambulate during labor	70.99	64.84	82.37(77.54–86.55)	49.38(41.39–57.38)	71.21	0.66(0.61–0.70)	1.10	Both
Women encouraged/assisted to drink liquids or eat during labor	86.59	75.82	91.30(87.8–94.06)	17.27(10.73–25.65)	89.23	0.63(0.58–0.69)	1.18	Both
Privacy provided during clinical care	78.24	64.84	82.71(77.90–86.85)	30.0(23.02–37.74)	78.24	0.64(0.58–0.68)	1.21	Both
Provider spent enough time with the mother	70.99	65.93	80.67(75.74–84.98)	44.52(36.54–52.70)	72.09	0.62(0.57–0.66)	1.09	Both
Provider introduced self to women	54.29	23.96	81.65(73.09–88.42)	54.34(48.92–59.67)	54.28	0.68(0.63–0.72)	2.27	AUC
What will happen to women in labor was explained at least once	54.95	30.33	71.74(63.45–79.07)	49.84(44.20–55.48)	56.71	0.61(0.56–0.65)	1.87	AUC
Women were encouraged to assume different positions during labor	56.04	36.26	74.55(67.19–81.01)	53.10(47.18–58.96)	56.93	0.63(0.58–0.68)	1.57	AUC
Providers responded professionally when women asked for help	94.95	88.13	97.26(95.14–98.62)	20.37(10.63–33.53)	95.17	0.58(0.53–0.63)	1.08	IF
Provider did not physically abuse the women	87.47	81.32	88.92(85.27–91.93)	18.82(11.16–28.76)	87.47	0.61(0.54–0.67)	1.08	IF
Provider did not abandon patient without care	61.32	81.76	61.83(56.68–66.79)	40.96(30.28–52.31)	61.32	0.52(0.47–0.56)	0.75	IF
Provider demonstrated good communication, collaboration with clients and colleagues	76.48	87.25	91.95(88.58–94.59)	23.36(15.72–32.53)	90.00	0.56(0.52–0.61)	1.03	IF
Women provided oral consent before examination	49.89	37.80	60.47(52.74–67.82)	55.83(49.83–61.70)	50.33	0.58(0.54–0.63)	1.33	No
Women allowed to have a companion during delivery	23.74	18.90	23.26(14.82–33.61)	76.15(71.47–80.41)	23.74	0.50(0.45–0.54)	1.26	No
Women were not verbally abused	94.73	73.41	94.91(91.98–97.01)	5.79(2.36–11.56)	94.72	0.51(0.46–0.55)	1.29	No
Treat clients equally without discrimination	93.85	71.43	97.85(95.61–99.13)	16.15(10.29–23.63)	93.85	0.57(0.53–0.62)	1.31	No

Notes: True prevalence indicates observers’ prevalence

Criteria Met - Indicator meets either both AUC and IF, AUC only or IF only after validation analysis done

*IF* Inflation factor, *AUC* Area under receiver operating characteristic curve

### Content of care indicators

Of the 39 routine contents of child birth care signal function indicators, eighteen of them met both individual and population level acceptability criteria. For example: 40% of the women reported receiving the HIV test, which closely approximated the true prevalence of 44%. The validity analysis showed that women were able to accurately report whether they received an HIV test or not (SN: 94%, SP: 81%). Furthermore, the indicator of breastfeeding initiated within first hour of birth did meet both validity criteria. This indicator had high sensitivity (88%) and low specificity (17%), suggesting that while most women who initiate breast feeding in the first hour correctly reported doing so, nearly one out of eight women who did not breastfeed in the first hour falsely reported doing so (83%).

Ten indicators met the facility level of accuracy but over reported at the population level. These were: taking a urine sample for a protein test (AUC = 0.71, 95% CI: 0.67–0.75), recording blood pressure at the first post-delivery exam (AUC = 0.66, 95% CI: 0.61–0.70), taking a woman’s temperature at the first post-delivery exam (AUC = 0.74, 95% CI: 0.70–0.78), asking a women if she needed pain relief medication during labor (AUC = 0.75, 95% CI: 0.71–0.79), whether the health care provider discussed self-care and other healthy behaviors (AUC = 0.67, 95% CI: 0.63–0.72), counseling of women on consuming a balanced diet (AUC = 0.64, 95% CI: 0.60–0.69), whether the provider discussed delaying the baby’s bath until 24 h post-birth (AUC = 0.66, 95% CI:0.61–0.70), PNC appointment counseling (AUC = 0.69, 95% CI: 0.65–0.73), a composite indicator of 9 elements of immediate postpartum care counseling (AUC = 0.61, 95% CI: 0.54–0.65) and four basic items of immediate postpartum care counseling (AUC = 0.69, 95% CI: 0.65–0.73).

Nine content of care indicators met the acceptability criteria of the population level. These were: the provider asked about a woman’s obstetric history (AUC = 0.57, 95% CI: 0.53–0.62, IF: 1.24), a woman’s HIV status was checked (AUC = 0.59, 95% CI: 0.54–0.63, IF: 1.18), an abdominal examination was performed (AUC = 0.55, 95% CI: 0.50–0.59, IF: 1.04), the health care provider wore sterile gloves (AUC = 0.53, 95% CI: 0.48–0.58, IF: 1.14), the newborn was dried and wrapped with towel (AUC = 0.52, 95% CI: 0.47–0.57, IF: 1.17), a safe and clean environment was provided (AUC = 0.56, 95% CI: 0.51–0.60, IF: 1.06), the baby was weighed (AUC = 0.59, 95% CI: 0.55–0.64, IF: 0.96), the provider discussed perineum care (AUC = 0.58, 95% CI: 0.54–0.63, IF: 1.24) and a composite indicator of 5 essential elements of newborn care (AUC = 0.56,95% CI:0.52–0.61, IF: 0.83). Most of these valid indicators had low specificity, indicating there is high false positive rate. For example, only 6.0% of the women correctly reported that the health care provider didn’t wear sterile gloves during vaginal examination. However, a composite indicator of 5 essential elements of newborn care had low sensitivity (38%) and high specificity (76%) indicates a high false negative rate, which shows, 62% of woman who did receive all the five elements of newborn care did not report receiving those interventions.

Of the content of care indicators which did not meet either of the validity criteria were: the scale was calibrated and the baby was weighed (AUC = 0.48 ± 0.05, IF: 0.72) and a women’s vulva was cleansed (AUC = 0.59 ± 0.04, IF: 1.99) which showed the observed prevalence was nearly double at the population level compared to the facility where data were collected for this study [Table 4].

**Table 4:** Validation Results on the Content of Child Birth Care Quality indicators in Northern Ethiopia (*N* = 455)

Indicators	Women’s reported prevalence (%)	True prevalence (%)	Sensitivity of women’s self-report (95% CI)	Specificity of women’s self-report (95% CI)	Population survey estimate (Pr)	AUC (95% CI)	IF	Criteria met
HIV test offered	23.93	34.43	92.56(66.13–99.82)	93.18(81.34–98.57)	36.34	0.92(0.81–0.97)	1.06	Both
Received HIV test	40.17	44.26	93.75(69.77–99.84)	80.95(65.88–91.40)	52.11	0.87(0.77–0.95)	1.18	Both
Took temperature during labor	62.64	55.38	87.70(82.99–91.49)	66.50(59.56–72.96)	63.52	0.77(0.73–0.81)	1.15	Both
Took blood pressure at labor	83.96	76.92	91.71(88.32–94.38)	36.19(27.04–46.15)	85.27	0.62(0.58–0.67)	1.11	Both
Provider washed hands with soap/ antiseptic before initial examination	36.70	33.85	53.25(45.05–61.32)	70.11(64.58–75.22)	37.80	0.64(0.59–0.68)	1.12	Both
Vaginal examination performed	96.26	94.07	97.20(95.15–98.54)	14.81(4.19–33.73)	96.49	0.64(0.54–0.70)	1.03	Both
Correct uterotonic received, 1–3 min after birth	79.34	75.38	80.47(75.87–84.53)	16.07(9.81–24.21)	81.32	0.61(0.56–0.67)	1.08	Both
First post-delivery exam, bleeding checked	80.44	77.58	87.54(83.63–90.80)	41.18(31.52–51.36)	81.10	0.64(0.60–0.69)	1.05	Both
Women received pain relief medication	22.42	19.78	60.0(49.13–70.19)	84.93(80.84–88.44)	23.96	0.73(0.69–0.77)	1.21	Both
Clamped and cut cord of newborn when pulsations stopped	68.13	59.78	66.18(60.22–71.78)	27.32(21.0–34.39)	68.79	0.60(0.52–0.64)	1.15	Both
Baby placed immediately skin-to-skin on mother	81.98	80.22	87.12(83.25–90.38)	30.0(20.79–40.57)	83.73	0.60(0.54–0.65)	1.04	Both
Breast feeding initiated within first hour of birth	86.15	76.04	88.44(84.59–91.61)	17.43(10.83–25.87)	87.01	0.63(0.57–0.69)	1.14	Both
Newborn received tetracycline eye ointment	59.78	72.53	72.73(67.58–77.46)	70.4(61.58–78.23)	60.88	0.72(0.68–0.76)	0.84	Both
Two elements of essential newborn care (place skin to skin, & breastfed within first hour)	71.21	61.76	77.58(72.25–82.32)	39.08(31.79–46.75)	71.21	0.60(0.54–0.65)	1.15	Both
Provider discussed exclusive breast feeding	78.24	69.67	88.33(84.27–91.65)	43.48(35.07–52.18)	78.68	0.65(0.61–0.70)	1.13	Both
Provider discussed birth spacing	64.62	62.2	84.81(80.1–88.78)	68.6(61.1.-75.45)	64.60	0.77(0.73–0.81)	1.04	Both
Provider discussed immunization and other prophylaxis	76.48	62.2	85.87(81.25–89.71)	37.79(30.52–45.49)	76.93	0.61(0.57–0.66)	1.24	Both
Provider discussed and reviewed possible complications and readiness plan for mother and newborn	67.91	58.46	81.95(76.80–86.38)	50.79(43.44–58.12)	68.35	0.67(0.63–0.71)	1.17	Both
Provider took urine sample for protein test	36.48	24.18	69.09(59.57–77.55)	73.33(68.33–77.93)	36.93	0.71(0.67–0.75)	1.53	AUC
At the first post-delivery exam, provider took blood pressure	66.15	52.53	83.68(78.37–88.13)	49.54(42.68–56.40)	67.91	0.66(0.61–0.70)	1.29	AUC
At the first post-delivery exam, provider took temperature	49.67	35.38	80.75(73.80)	65.99(60.26–71.39)	50.55	0.74(0.70–0.78)	1.43	AUC
Women asked if they needed pain relief medication	26.15	19.78	66.67(55.95–76.26)	81.64(77.28–85.48)	27.92	0.75(0.71–0.79)	1.41	AUC
Provider discussed self-care and other healthy behaviors	59.78	46.59	79.25(73.16–84.50)	55.97(49.48–62.31)	60.44	0.67(0.63–0.72)	1.30	AUC
Women counseled on balanced diet	53.41	35.16	72.5(64.89–79.25)	56.27(50.40–62.01)	53.85	0.64(0.60–0.69)	1.53	AUC
Provider discussed delayed baby bath until 24 h	52.31	41.32	73.94(67.05–80.05)	59.93(53.78–65.85)	54.05	0.66(0.61–0.70)	1.31	AUC
Provider discussed schedule of next PNC visit	58.46	40.66	81.62(75.28–86.92)	56.30(50.15–62.30)	59.12	0.69(0.65–0.73)	1.45	AUC
4 elements of care provision discussed (EBF, birth spacing, immunization and review complication and readiness plan)	45.27	29.67	77.04(69.02–83.83)	68.13(62.71–73.20)	45.27	0.69(0.65–0.73)	1.53	AUC
Asked about obstetric history	88.57	71.65	92.33(88.89–94.98)	20.93(14.27–28.97)	88.57	0.57(0.53–0.62)	1.24	IF
HIV status checked	78.68	66.59	84.49(79.91–88.37)	32.89(25.50–40.97)	78.68	0.59(0.54–0.63)	1.18	IF
Abdominal examination performed	96.92	93.85	97.89(96.04–99.03)	14.29(4.03–32.67)	97.14	0.55(0.50–0.59)	1.04	IF
Health provider wore sterile gloves for vaginal examination	92.31	80.88	92.12(88.88–94.66)	5.75(1.89–12.90)	92.53	0.53(0.48–0.58)	1.14	IF
Newborn immediately dried and wrapped with towel/ cloth	87.47	75.16	88.01(84.09–91.26)	11.50(6.27–18.87)	88.12	0.52(0.47–0.57)	1.17	IF
Five elements of essential newborn care (dried, skin to skin, clamped cord, breastfed within first hour and received tetracycline ointment)	27.69	22.86	38.46(29.09–48.51)	75.50(70.65–79.91)	18.90	0.56(0.52–0.61)	0.83	IF
The baby was weighed	83.96	87.25	85.86(82.03–89.14)	32.20(20.62–45.64)	83.56	0.59(0.55–0.64)	0.96	IF
The provider ensured a safe and clean care environment for women	83.96	79.12	86.39(82.41–89.76)	24.21(16.0–34.08)	84.18	0.56(0.51–0.60)	1.06	IF
Provider discussed perineal care	70.99	57.36	77.01(71.42–81.97)	36.60(29.81–43.80)	71.21	0.58(0.54–0.63)	1.24	IF
The scale was calibrated and the baby was weighed	35.48	56.65	47.51(40.77–54.31)	30.82(23.75–38.62)	40.89	0.48(0.43–0.53)	0.72	No
Women’s vulva was cleansed	52.75	31.21	65.49(57.06–72.26)	39.62(34.16–45.27)	61.97	0.59(0.54–0.63)	1.99	No

**Notes:** True prevalence indicates observers’ prevalence

Criteria Met Indicator met either both AUC and IF, AUC only or IF only after validation analysis done

*IF* Inflation factor, *AUC* Area under receiver operating characteristic curve

### Non-indicated childbirth care practice indicators

Of the 8 non-indicated care practices during normal birth, 2 indicators (stretching of the perineum during second stage of labor (AUC = 0.60 ± 0.05, IF: 1.16) and episiotomy performed without indication (AUC = 0.60 ± 0.05, IF: 1.09)) met both validity criteria.

Five indicators met the individual-level accuracy: Apply fundal pressure (AUC = 0.76, 95% CI: 0.72–0.80), artificial rupture of membrane (AUC = 0.67, 95% CI: 0.62–0.71), restriction of foods and fluids (AUC = 0.64, 95% CI: 0.61–0.69), frequency of digital vaginal examination less than four hours (AUC = 0.67, 95% CI: 0.62–0.71) and routine intravenous fluid infusion during labor (AUC = 0.74, 95% CI: 0.69–0.78). One indicator of the non-indicated care practices, which is hold newborn upside down did not meet either validation criteria (AUC = 0.55 ± 0.05, IF: 3.02) [Table 5].

**Table 5:** Validation Results for Non indicated Childbirth Care Practice Quality indicators in Northern Ethiopia (*N* = 455)

Indicators	Women’s reported prevalence (%)	True prevalence (%)	Sensitivity of women’s self-report (95% CI)	Specificity of women’s self-report (95% CI)	Population survey estimate (Pr)	AUC (95% CI)	IF	Criteria Met
Stretching of perineum during second stage of labor	14.51	13.63	33.87(22.33–47.00)	87.02(83.29–90.18)	15.83	0.60(0.56–0.65)	1.16	Both
Episiotomy performed without indication	25.49	24.62	37.5(28.53–47.15)	76.68(71.84–81.05)	26.82	0.60(0.55–0.65)	1.09	Both
Apply fundal pressure to hasten delivery	6.15	4.18	57.89(33.50–79.74)	95.18(92.73–96.99)	7.04	0.76(0.72–0.80)	1.68	AUC
Artificial rupture of membrane	6.59	5.27	41.67(22.11–63.35)	93.04(90.21–95.25)	8.79	0.67(0.62–0.71)	1.67	AUC
Restriction of foods and fluids	14.95	7.69	37.14(21.47–55.08)	85.48(81.74–88.70)	16.26	0.64(0.61–0.69)	2.11	AUC
Digital vaginal examination less than four hours frequency	22.64	17.36	51.90(40.36–63.29)	80.32(75.93–84.22)	25.27	0.67(0.62–0.71)	1.46	AUC
Routine intravenous fluid infusion for all laboring women	31.65	15.82	72.22(60.41–82.14)	75.46(70.83–79.69)	32.09	0.74(0.69–0.78)	2.03	AUC
Hold newborn upside down	3.96	4.62	33.87(22.33–47.00)	87.02(83.29–90.18)	13.95	0.55(0.50–0.60)	3.02	No

**Notes:** True prevalence indicates observers’ prevalence

Criteria Met - Indicator met either both AUC and IF, AUC only or IF only after validation analysis done

*IF* Inflation factor, *AUC* Area under receiver operating characteristic curve

### Maternal and newborn complications

#### Maternal complications

Participants were questioned about whether they experienced any of the following conditions either during or immediately following delivery: (1) bleeding, (2) preeclampsia/eclampsia (3) laceration (4) another type of complication (asked to specify), or (5) no complications.

Indicators of women’s report of experiencing any type of complication, hemorrhage, laceration and avoiding delays in received care met both validity criteria. About reporting the prevalence of maternal complications, nearly 19% of women reported experiencing some type of complication, which exceeded the observed prevalence (15%). Self-reports of experiencing any complication had a sensitivity of 45%, indicating that around half of women who had experienced a complication did report it. The indicator also had high specificity (85%), reflecting a low rate of false positive reports by women. The indicator of avoiding delays in receiving care had a high specificity (91.82, 95% CI: 88.64–94.33) but low sensitivity (32.81, 95% CI: 21.59–45.69). In addition, the most commonly reported indicators by mothers were experiencing excessive hemorrhage (9.67%), followed by laceration (3.96%).

Three indicators met the individual-level accuracy: Preeclampsia/eclampsia, neonatal complication and new born death within the facility. The indicator of preeclampsia/eclampsia faced around birth had low sensitivity (38%) and high specificity (95%) and was accurately classified at individual level (AUC = 0.62, 95% CI: 0.53–0.70). This shows there is a high false negativity rate and an overestimation at the population level (IF = 1.5).

#### Newborn outcomes

Mothers were asked whether their newborn babies were faced with any of the following complications during birth: (1) birth asphyxia, (2) still birth (3) infection (4) newborn death within the health facility (5) any other type of complication, or (6) no complications.

Only the birth asphyxia indicator of the newborn complication met both validity criteria (AUC = 0.76, 95% CI: 0.68–0.84, IF: 1.19). Women’s reports on birth asphyxia had a sensitivity of 64%, indicating that over one-third of women who had asphyxiated newborns did not report it. However, the indicator had high specificity (95%), reflecting low false positive reports.

The indicator of any neonatal complication only met study validity criteria at the individual level (AUC = 0.71, 95% CI (0.68–0.84), IF: 1.39), but suggests that the indicator was overestimated by 1.39 at the population level. Likewise, the indicator facility newborn death met individual-level accuracy (AUC = 0.60, 95% CI (0.45–0.73), IF: 0.47). This indicates that the indicator was underestimated by 0.47 at the population level. Implies the indicator had low sensitivity and high specificity indicating not all women whom their newborns death at facility correctly reported it. This might be due to mothers unable to differentiate newborn death and still birth.

Only the still birth indicator did not meet either of the validation criteria (AUC = 0.57 ± 0.07, IF = 1.52). Regarding the perinatal death (still birth and neonatal death) indicator, mothers could not differentially report whether the death was a still birth or early newborn death.

Lastly this study revealed that, 17% of women reported their newborns suffered at least one type of complication, exceeding the observed prevalence (12%) [Table 6].

**Table 6:** Validation Results on Maternal and Newborn Outcome Quality Indicators in Northern Ethiopia (*N* = 455)

Indicators	Women’s reported prevalence (%)	True prevalence (%)	Sensitivity of women’s self-report (95% CI)	Specificity of women’s self-report (95% CI)	Population survey estimate (Pr)	AUC (95% CI)	IF	Criteria met
Maternal obstetric complication (yes to any)	18.68	14.95	45.59(33.45–58.12)	85.79(81.91–89.11)	17.90	0.65(0.61–0.70)	1.19	Both
Severe bleeding (hemorrhage)	9.67	8.35	28.95(15.42–45.90)	92.32(89.34–94.69)	9.46	0.60(0.53–0.66)	1.13	Both
Tear/laceration in vagina	3.96	3.52	50.0(24.65–75.35)	97.72(95.85–98.90)	3.96	0.74(0.69–0.78)	1.12	Both
Delays were not experienced during care	11.65	14.07	32.81(21.59–45.69)	91.82(88.64–94.33)	11.65	0.64(0.57–0.71)	0.83	Both
Birth asphyxia	8.13	6.81	64.52(12.76–64.86)	95.99(93.66–97.65)	8.13	0.76(0.68–0.84)	1.19	Both
Preeclampsia, eclampsia	5.93	3.96	38.89(17.30–64.25)	95.42 (93.02–97.18)	5.94	0.62(0.53–0.70)	1.50	AUC
Neonatal complication (yes to any)	17.36	12.53	64.91(51.10–77.10)	89.45(86.00–92.29)	14.34	0.71(0.65–0.76)	1.39	AUC
Newborn death within facility	1.98	4.18	10.53(1.30–33.14)	98.42(96.72–99.35)	1.95	0.60(0.45–0.73)	0.47	AUC
Still birth	6.37	4.18	26.32(9.15–51.20)	94.50(91.92–96.44)	6.37	0.57(0.50–0.64)	1.52	No

Notes: True prevalence indicates observers’ prevalence

Criteria Met - Indicator met either both AUC and IF, AUC only or IF only after validation analysis done

*IF* Inflation factor, *AUC* Area under receiver operating characteristic curve

## Discussion

This study tested the validity of key indicators that measure the quality of care received at the time of the intra-partum and immediate postpartum period which is needed to move beyond measures of nominal facility utilization for delivery to measures of effective coverage of delivery care that are either currently in use or will be incorporated into the household survey. Measures of effective coverage weight utilization estimates by the quality of the services used [24].

Several (*n* = 32) of the quality indicators across phases of labor and delivery met both validity criteria (accuracy at AUC and IF). Furthermore, an additional 21 indicators met the individual-level criteria, 13 met the population level criteria and 8 indicators did not meet any of the criteria. Indicators that did not meet both criteria are not necessarily or invalid for all measurement purposes, but should be used in accordance with the rationale for their use [9, 25]. Indicators that met both validity criteria could be appropriate for the measurement of quality of care at the facility and population level.

Indicators that do not meet criteria for the AUC but do meet the IF criterion may be suitable to measure intervention coverage at the population-level as false positive and negative reporting balance each other at the aggregate level.

Indicators that do met AUC criteria, but not IF criteria may be useful for facility-level measurement or individual level classification but may be over reported at population level. For example, in our study the composite indicator of counseling on care provision of immediate postpartum care, taking a urine sample for a protein test indicator was not correctly reported by women at the population level but, accurate measurement occurred at the individual level because of some of the indicators required understanding of technical terms which are difficult to distinguish by mothers.

Our study shows varied results in specific indicators when compared with other published literature findings. For example the indicator of women being allowed to have a companion of her choice in labor found that most women accurately reported the presence of a companion (i.e., high sensitivity). Our result shows a low specificity for this indicator, which may reflect “facility reporting bias” among women. This finding is not consistent with a study done in Mozambique and Kenya [14, 21], that reported high specificity for the indicator of women being allowed to have a companion of choice during labor. This discrepancy could be attributable to the cultural contexts of mothers, literacy level and awareness on the importance of having a companion of choice during labor. Besides, Mothers may have under reported negative experiences at a facility due to concerns about providers abandoning proper care for their subsequent visit in retaliation for these comments." We also found low sensitivity and high specificity for reported excessive bleeding (hemorrhage); this finding corresponds with levels found among women delivering in Mexico, Indonesia, Benin and the Philippines respectively [9, 26–28]. However, these results differ from women’s reporting in Ghana [29]. Taken together, the findings suggest that women’s understanding and recall of the presence of a companion of choice and obstetric complications experienced may vary by clinical and cultural context, and settings. The study results indicate that there were challenges of measuring low prevalence indicators accurately. Given that the calculation of IF depends upon the indicator’s observed prevalence, even a small number of false positive responses can result in overestimation as measured by the IF. A few of indicators met the IF test only; individual–level misclassification does not inherently signify that measurement at the population level will be inaccurate [21, 30, 31]. Evidence shows that knowledge of whether an indicator is likely to be overestimated can also have significant programmatic implications. For example, where the complication of preeclampsia/eclampsia is over– reported, identifying causes of maternal death at population level may not be as great as expected. When possible, we recommend that users also triangulate self–reported data on quality of care with facility and other data sources. Other core indicators like calibrating the scale and weighing the baby did not meet any of the validity criteria. This suggests that women may not be able to report accurately when and where the measuring scale was placed and calibrated.

This study has some limitations. This validation results don’t include women indicated for a cesarean section delivery and mothers whom were referred to higher facility with surgical capacity. Furthermore, this tool is used at discharge (soon after labor) and considers population level indicators. It may have some deficiencies for household surveys that take place long after delivery.

## Conclusion

Women were able to accurately report on several aspects of quality of care indicators received across the phases of child birth and immediately after birth. A few technical indicators tended to have higher don’t know responses.

Although high specificity and sensitivity are preferred for all indicators, knowing the estimated survey-based prevalence is helpful, particularly for indicators of very low prevalence which are likely to be overestimated without near perfect specificity. Likewise, in some cases, low sensitivity and specificity cancel out at the population level and may generate acceptable estimates for coverage monitoring purposes, even if they are not appropriate for analysis at the individual or facility level. Therefore, the valid indicators should be included as potential measurements of quality process of labor and delivery to ensure that the essential content of care interventions is delivered.

Additional work on fewer, better metrics of quality measurement indicators of mothers and newborns through national survey efforts and tracking the basic lifesaving interventions received at the time of birth are reasonable to design a range of context-based quality improvement strategies. Lastly, the valid indicators can be tracked and reported through the health management information system or district health information system two (DHIS2) data for decision making at all levels.

## Supplementary information

**Additional file 1: Appendix 1**. Full List of Indicators on Quality of Care during intra-partum and immediate postpartum period in Northern Ethiopia

**Additional file 2: Appendix 2**. Check list or QuestionnaireR

## Data Availability

The raw datasets used and/or analyzed during the current study are available from the corresponding author on reasonable request.

## Abbreviations

HCs: Health care providers
SN: Sensitivity
SP: Specificity
