# Supplementary material for: Beyond health system contact: measuring and validating quality of childbirth care indicators in primary level facilities of northern Ethiopia
Source: Reprod Health. 2020 May 24;17:73. doi: 10.1186/s12978-020-00923-w (PMC7247130; doi:10.1186/s12978-020-00923-w)
Supplement: Supplementary file 1 — Additional file 1: Appendix 1. Full List of Indicators on Quality of Care during intra-partum and immediate postpartum period in Northern Ethiopia [file 12978_2020_923_MOESM1_ESM.docx]

**Appendix: Full List of Indicators on Quality of Care during intra-partum and immediate postpartum period in Northern Ethiopia**

| *List of Indicators by category* |
| --- |
| *Respectful maternity care indicators* |
| Women & her companion greeted respectfully |
| Provider introduced to women |
| Actively listened to the women |
| Women allowed to have companion choice in labor |
| Women obtained oral consent before examination |
| At least once, explained what will happen in labor to woman |
| Encourages women to ambulate during labor |
| Encourages woman to assume different positions during labor |
| Encourages/assists the woman to drink liquids or eat |
| Women allowed to have companion during delivery |
| Privacy in providing clinical care |
| Did not verbally abuse |
| Treat clients equally without discrimination |
| Providers responds professionally when women ask for help |
| Time the provider spent with women |
| Did not physically abuse clients |
| Did not abandon patient without care |
| Good communication, collaboration with clients and colleague |
| *Content of care indicators* |
| Ask obstetric history |
| HIV status checked |
| HIV test offered |
| Woman received HIV test |
| Takes temperature during labour |
| Takes blood pressure at labour |
| Takes urine sample for protein |
| Abdominal examination performed |
| Provider’s washes hands with soap & water or antiseptic before initial examination |
| Health provider wore sterile gloves for vaginal examination |
| Women’s vulva cleansed |
| Vaginal examination performed |
| Correctly uterotonic received, 1-3 minute after birth |
| First post-delivery exam, bleeding checked |
| First post-delivery exam, takes blood pressure |
| First post-delivery exam, provider takes temperature |
| Women asked for pain relief medication at some time |
| Women received pain relief medication |
| Newborn immediately dried & wrapped with towel/ cloth |
| Clamped and cut cord of newborn when pulsations stop |
| Baby place immediately skin-to-skin on mother |
| Breast feeding initiated with in first hour of birth |
| Newborn received TTC eye ointment |
| 2 elements of essential newborn care (Place skin to skin, and breastfed within first hour) |
| Baby weighed |
| Scale calibrated & baby weighed |
| Ensured safe and clean care environment for women |
| Discussed on Perinal care |
| Discussed on exclusive breast feeding |
| Discussed on birth spacing |
| Discussed on immunization and other prophylaxis |
| Discussed on self-care & other healthy behaviors |
| Counseled on balanced diet |
| Discussed on delayed baby bath until 24 hours |
| Discussed & reviewed possible complication and readiness plan for mother & newborn |
| Discussed on schedule the next PNC visit |
| Discussed & counseled on 9 elements of care provision of immediate postpartum care |
| 4 elements of care provision discussed (EBF, birth spacing, immunization & review complication & readiness plan) |
| After the delivery of your baby, did the provider measure the Apgar score within 1st and 5th minutes? |
| Was your baby weighed and did the provider tell you the weight of your baby? |
| While you were in the health facility for the birth of your baby, did the provider record the activities done? |
| Did the health care provider monitor your progress of labor through Parthograph? |
| Immediately after your placenta was expelled, did the provider examine it? |
| Did the provider dry the cord or use of Chlorhexidine? |
| Did the provider apply controlled cord traction? |
| After you gave birth, did anyone give your baby an injection called “vitamin K”? |
| After your stay in the postnatal room, were you checked by a senior staff member of the facility before you were discharged? |
| When you were transferred to the delivery room, did you find the delivery coach/bed was clean? |
| Did the provider take your pulse during your labor? |
| Did the provider perform rapid initial assessment when you arrived to health facility? |
| After you gave birth, did anyone check your perineum for any kind of laceration? |
| Did the provider palpate your uterus 15-minute following delivery of the placenta? |
| Did the provider record the birth weight of the newborn |
| *Non indicated obstetrics care practices indicators* |
| Use of enema |
| Pubic shaving |
| Slapping the newborn |
| Something other than breast milk given to the baby in the first hour of birth |
| Apply fundal pressure to hasten delivery |
| Hold newborn upside down |
| Stretching of perineum during second stage of labor |
| Artificial rupture of membrane |
| Restriction of foods & fluids |
| Digital vaginal examination less than four hours |
| Episiotomy performed without indication |
| Routine intravenous fluid infusion for all laboring women |
| *Maternal and Newborn Outcomes indicators* |
| Maternal obstetric complication (yes to any) |
| Sever bleeding (hemorrhage) |
| Preeclampsia, eclampsia |
| Tear/laceration |
| Delays in received care |
| Neonatal complication(yes to any) |
| Birth asphyxia |
| Still birth |
| New born death within facility |
