# Supplementary material for: Beyond health system contact: measuring and validating quality of childbirth care indicators in primary level facilities of northern Ethiopia
Source: Reprod Health. 2020 May 24;17:73. doi: 10.1186/s12978-020-00923-w (PMC7247130; doi:10.1186/s12978-020-00923-w)
Supplement: Supplementary file 2 — Additional file 2: Appendix 2. Check list or QuestionnaireR [file 12978_2020_923_MOESM2_ESM.docx]

**መቐለ ዩኒቨርሲቲ ጥዕና ሳይንስ ኮሌጅ ሕብረተሰብ ት/ቤት**

**መምርሒ: ቅጥዒ ሓበሬታ መውሃቢ ፍቃድ መውሰድን ንኣዴታት ኣብ እዋን ምስንባት**

ዳሕና ዶ ውዒልለን ዝኸበራኣደ!

ጥዕና ይሃበለይ፣ ሽመይ­­ ___________ ይበሃል፡፡ ንሕና ኣብ ሰሜን ኢትዮጵያ ኣብ ዝርከባ ጥዕና ትካላት ብዛዕባ ኣወሃህባ ፅሬት ግልጋሎት ወሊድን ቅፅበታዊ ክንክን ድሕረ ወሊድን መፅናዕቲ ክነካይድ ኢና፡፡ ዕላማ ናይዚ ትዕዝብታዊ/ቓለ መሕትት መፅናዕቲ ድማ ኣብ ግዘ ወሊድን ቅፅበታዊ ክንክን ድሕረ ወሊድንዝወሃብ ግልጋሎት ፅሬቱ ምልካዕ እዩ፡፡ስለዚ እዚ ሓበሬታ ንምእካብ ፣ ንሰን ኣካል ናይዚ መፅናዕቲ ንኽኾና ሓሪና ዓዲምናየን ኣለና፡፡ ፍቓደኛ እንድሕር ኮይነን ፣ ብዛዕባ እቲ መፅናዕቲ ከረድአን እሞ ኣብቲ ፍቓድ መሕተቲ ዝተዳለወ ቅጥዒ ክፍርማለይ እየን፡፡ ልክዕ እዩኣነ እቲ ኣብ ግዘ ወሊድን ድሕረ ወሊድ ቅፅበታዊ ክንክንድሕረ ወሊድንዝግበረለንግልጋሎትን ንምርኣይ ኣብቲ መዋለዲ ክፍሊ ስለዘለኹ ስኽፍ ክብለን ይኽእል እዩ፣ ኮይኑ ግናብዙሕ ኣይሕሰባ፣ ኣነ ባዕለይ በዓል ሞያ ጥዕናን፣ ቅድሚ ሕጂ ኣብ ዝተፈላለዩ ጥዕና ትካላት ግልጋሎት ወሊድ ዝህብ ዝነበርኩን እየ፡፡ ዉፅኢት ናይዚ መፅናዕቲ ብቀጥታ ወይ እዉን ብተዘዋዋሪ ንባዕለንን ንቤተሰበንን ዝህቦ ረብሓ ኣዚዩ ዝለዓለ እዩ፡፡ ምኽንያቱ ንቐፃላይ ቤተሰበን ወይ እዉን ባዕለን ተመሳሳሊ ግልጋሎት ክረኽባ ስለዝኽእላ ማለት እዩ፡፡ ኣብዚ ፅንዓት ብምስታፈን ዝኽፈለን ገንዘብ ኮነ ካሊእ ነገር የለን፡፡ እቲ ዝሃባኒ ሓበሬታ ምስጢራዉነቱ ኣዝዩ ዝተሓለወ እዩ፣ ብተወሳኺ እዉን እቲ ሓበሬታ ዝእክበሉ ዘለኹ ወረቐት ሽመን ከይፀሓፈሉ ኣብ ኮመዲኖ ብቑልፊተቖሊፍ እዩ ክቕመጥ፡፡ኮይኑ ግና መለለዪ ኮድ ክግበረሉ እዩ፡፡ ኣብ ዝኮነ ይኩን እዋን ኣብቲ መፅናዕቲ ናይዘይምስታፍ ምሉእ መሰለን እውን ዝተሓለወ እዩ፣ ኣብዚ ፅንዓት ኣይሳተፍን ብምባልን ኣብቲ ትካል ምስ ዝግበረለን ግልጋሎት ጥዕና ምንም ረኽቢ የብሉን / ብዘይ ምስታፈን ዝበፅሐን በደል ሓንቲ እውን የለን፡፡ ብዛዕባ እቲ መፅናዕቲ ክሓተኦ ዝደልያ ዝኾነ ዓይነትሕቶ እንተሃሊወን ክሓታ ይክእላ እየን፡፡ ሕቶ ኣለወን ድዩ?

ንዝበለፀ ሓበሬታ እንተድልይወን ወይ ድማ በዚ ፅንዓት ምክንያት ፅገም እንተጋጢምወን ፣ ካብ ዋና ተማራማራይ ናይዚ መፅናዕቲ፣ ተምሃራይ ፒኤችዲ (ሳልሳይ ዲግሪ) ዝኾነ ሃፍቶም ገብረሂወት ወ/ኣረጋይ ስልኪ ቁፅሪ (0910209985) ወይ ድማ መቐለ ዩኒቨርስቲ ኮለጅ ሳይንስ ጥዕና፣ ናይቲ ትካል ስነ ምግባር ፅንዓትን ምርምርን ቦርድስልኪ ቁፅሪ (0344410103) ብምድዋል ክረክባ ይክእላ እየን፡፡

**ቅጥዒ መሕተት ፍቓድ መርከቢ፡** በይዘኦምሓደ ቅዳሕ ፍቓድ መሕተቲ ቅጥዒ ንተሳተፍቲ ይሃብወን ወይ ድማ ብደንቢ የረድእወን፡፣ አብዚፅንዓትንኽካፈልብአጋጣሚዝተመርፅኩሰለዝኾንኩአብዚፅንዓትብዝገብሮተሳትፎብሙሉእፍቓደይከምዝኸነን፤ቅጥዑምምላእይኹንምግዳፍከምዝኽእልን፤ዕላማ ናይቲ መፅናዕቲ ብዉነ ስለ ዝተረደኣኒ፣ ኣብቲ ፅንዓት ንኽሳተፍ ፍቓደኛ እየ?

እወ ይቐፅሉ ፣ ኣይፋለይን ምሕታት የቛርፁ

ናይ ተሳታፊት (ኣዶ) ፌርማ_____________

ሽም ሓበሬታ ኣካባይ_____________________ፌርማ_____________ ዕለት______/_____/______

ሽም ተቖፃፃሪ ናይቲ መፅናዕቲ_________________ፌርማ____________ ዕለት_____/______/________

**ንምትሕብባርክን ኣቕዲመ ካብ ልበይ የመስግን !!**

| **Identification Sheet** (መለለይ ናይቲ ቃለ መሕትት) |
| --- |
| H1፡Facility Name /ሽም ናይቲ ጥዕና ትካል፣__________________________ |
| H2: Facility Type / ዓይነት ናይቲ ጥዕና ትካል: Health center/ ጣብያ ጥዕና ………1  Hospital/ ሆስፒታል ……………2 |
| H3: Woreda’s Name/ ሽም ናይቲ ወረዳ ፡­­­­­­_______________________________ |
| H4: Total population of the Woreda/ በዝሒ ህዝቢ ናይታ ወረዳ፤ |
| H5: Catchment Health center population/ በዝሒ ህዝቢ ናይ ክላስተር፤ |
| **INSTRUCTION (መምርሒ)** |
| 1. Meet the facility manager/maternity head and explain the purpose, process and your roles during data collection and obtain permission/ ንመራሒ ናይቲ ትካል ወይም ሓላፊ ክፍሊ መዋልዳን ረኺብካ ፣ ብዛዕባ ዕላማ ናይቲ መፅናዕቲ፣ ከይዲ ናይቲ መፅናዕቲ፣ ግደ መፅናዓይ ኣብ እዋን ሓበሬታ ምእካብን ገለፃ ምግባርን ፍቓድ ምርካብን |
| 1. Along with facility manager and unit coordinator identify and include all SBAs (skilled birth attendants) in primary health facilities who are on the job at the time your observation/ምስ መራሒ ናይቲ ትካልን ወይ ሓላፊ መዋልዳን ክፍልን ብምኳን ንዝሰልጠኑ ሰብ ሞያ ጥዕና ኣብ ግዜ መፅናዕቲ ኣብ ስራሕ ንዝርከቡ ምርካብን ምልላይን |
| 1. Explain the purpose, process and your roles during data collection and obtain permission from both service provider and client/ ብዛዕባ ዕላማን ከይዲ ናይቲ መፅናዕትን፣ ግደ መፅናዓይን ነቶም ሰብሞያ ጥዕናን ተገልገልትን ገለፃ ብምግባር ፍቓድ ምርካብ፡፡ |
| 1. Read the consent paper to the service provider and client to be observed and ask for their permission to be observed (consent paper is attached)/ እቲ ፍቓድ መፅናዕቲ መርከቢ ቅጥዒ ዝሓዘ ወረቐት ንሰብ ሞያ ጥዕናን (ሓካይምን) ተሓከምትን ይንበበሎም እሞ፣ ተረዲእዎም ፍቓደኛታት ምኳናም ብምሕታት ምርግጋፅ፡፡ |
| 1. Take necessary infection prevention measures when observing the provider - client interactions/ ኣብ ግዜ ትዕዝብታዊ ሓበሬታ ምስብሳብ፣ ኣብ ሞንጎ ግልጋሎት ወሃባይንበዓል ሞያን ተሓከምትን ዘሎ ምስ ምክልካል ረኽስታት ዝተተሓዘ ርክብ ብምርኣይ ኣድላዪ ስጉምቲ ንኽዉሰድ ግበር፡፡ |
| 1. Complete all items by direct observation of the care and rate the care services of each task by circling 1 for “Yes” or 0 for “No”/ ኩሎም እቶም ብቐጥተዊ ትዕዝብቲ ኣብ ኣወሃህባ ግልጋሎት ወሊድን ቕፅበታዊ ዝወሃብ ክንክን ድሕረ ወሊድን ክምልኡ ዝተዳለዉ ነገራት መሊእኻ ፣ በቲ ሲዒቡ ተቐሚጡ ዘሎ ቁፅሪ ምደባ ግበረሎም፡፡   1= Yes = Observed or completely and correctly performed the activity by the provider  እወ፣ እቲ በዓል ሞያ ኣብቲ ዝተዓዘብካዮ ስራሕ ኩሉ ወይ ብዘይ ስሕተትእንተሰሪሕዎ  0= Not = Observed/ done incorrectly by the provider/ ብበዓል ሙያ ብጠቕላላ እንተዘይተሰሪሑን ወይ እዉን ብደንቢ እንተዘይኪኢልዎን |

**Part II –Factors related to labouring mother (ክፍሊ ክልተ፡ ምስ ኣደ ዝተትሓሓዙ ሕቶታት)**

| **S.No**  ተ.ቁ | **Questions/Variables**  ዝርዝር ሕቶ | **Circle answer from the responses**  ካብ ተሳተፍቲ ዝተረኽበ መልሲ ኣኽብብ | | | | **Skip to**  ናብ ቀፃሊ ሕቶ ይዘለል | |
| --- | --- | --- | --- | --- | --- | --- | --- |
| 201 | Maternal age (completed in years**)** /ዕድመ ኣደ(ብሙሉእ ዓመት) | _____________ year/ብዓመት ይገለፅ | | | |  | |
| 202 | What is the highest level of schooling you have ever attended?  ዝለዓለ ደረጃ ትምህርቲኪ እንታይ እዩ? | 1. Illiterate/ዘይተምሃረት 2. Read & write/ምንባብን ምፅሓፍን 3. Elementary school/ ቀዳማይ ደረጃ ዝወደአት(1ይ-8ይ ክፍሊ) 4. Secondary school/ ካልኣይ ደረጃ ዝወደአት(9ይ-12) 5. College/University**/** ኮለጅ/ዩኒቨርስቲ ዝወደአት | | | |  | |
| 203 | What is your marital status?  (ኩነታት ሓዳር) | 1. Married /ዝተመርዓወት 2. Single/never married/ዘይተመርዓወት/ ተመርያ ዘይትፈልጥ/ 3. Divorced /separated/ዝተፋትሐት 4. Widowed/ ሰብኣያ ዝሞታ | | | |  | |
| 204 | What is your occupation? ( ኩነታት ስራሕ) | 1. House wife/ልእልቲ ገዛ/በዓልቲ እንደ (ናይ ገዛ ስራሕ እትሰርሕ) 2. Daily worker/መዓልታዊ ሰራሕተኛ 3. Government employee/መንግስታዊ ሰራሕተኛ 4. Private employee/ናይ ዉልቐ ሰራሕተኛ 5. Others (specify)/ ካልእ እንተሃልዩ ይገለፅ____________ | | | |  | |
| 205 | What is your religion? (ሃይማኖት) | 1. Orthodox Christian/ ኦርቶዶክስ ክርስትያን 2. Muslim/ ሙስሊም 3. Others (specify)/ ካሊእ ይገለፅ___________ | | | |  | |
| 206 | Residence (እትነብርሉ ቦታ) | 1. Rural/ ገጠር 2. Urban/ ከተማ | | | |  | |
| 207 | Average walking distance in minutes from your home to health center (ርሕቐት ብእግሪ ጉዕዞ ካብ መንበሪ ገዛ ናብቲ ቐረባኺ ዘሎ ጥ/ትካል ብማአኸላይ ሰዓት) | ___________Minute/ ደቒቓ | | | |  | |
| 208 | Parity / በዝሒ ወሊድ | ___________(in number)/ (ብቑፅሪ) | | | |  | |
| 209 | Gravidity / በዝሒ ጥንሲ | ___________(in number) /(ብቑፅሪ) | | | |  | |
| 210 | History of abortion / ዘጋጠመኪ በዝሒ ምንፅል ጥንሲ | ___________(in number)/ (ብቑፅሪ) | | | |  | |
| 211 | History of still birth / ክውለድ ከሎ ዝሞተኪ በዝሒ ህፅን | ___________(in number) /(ብቑፅሪ) | | | |  | |
| 212 | Had ANC for this pregnancy? ነዚ ጥንሲ እዙይ ክትትል ጥንሲ ገይርኪ ዶ ነይርኪ? | 1. No/ኣይገበርኩን 2. Yes/ እወ | | | |  | |
| 213 | If yes for Q212, how many times did you receive? ንሕቶ ቁፅሪ 212 መልሰን እወ እንተኾይኑ፣ ክንደይ ግዘ ተከታትልኪ? | __________visits (ብቑፅሪ)  8. Don’t know/ ኣይፈልጦን | | | |  | |
| 214 | If yes for Q212, where did you receive? ንሕቶ ቁፅሪ 212 መልሰን እወ እንተኾይኑ፣ ኣበይ ክትትል ጥንሲ ትገብሪ ነይርኪ? | 1. Government hospital/ መንግስታዊ ሆስፒታል 2. Government health center /መንግስታዊ ጥዕና ጣብያ 3. Government health post/መንግስታዊ ጥዕና ከላ 4. Private hospital/ናይ ግሊ ሆስፒታል 5. Private clinic/ናይ ግሊ ክሊኒክ 6. Other (specify)/ካሊእ(ይገለፅ)________________ | | | |  | |
| 215 | If yes for Q 212 does your last ANC visit was in this facility?  ንሕቶ ቁፅሪ 212 መልሰን እወ እንተኾይኑ፣ ናይ መወዳእታ ክትትል ጥንሲ ኣብዚ ጥዕና ትካል ዶ ትገብሪ ነይርኪ? | 1. No/ኣይፋሉን 2. Yes/ እወ | | | |  | |
| 216 | Had birth preparedness and complication readiness for this pregnancy/ ቅድመ ወሊድ ጥንቓቐ ከምኡ ውን ፀገማት ንከየጋጥሙኪ ዘውፅእክዮ ትልሚ ነይርኪ ዶ? | 1. No/ኣይፋሉን 2. Yes/ እወ | | | |  | |
| 217 | Have you ever heard about obstetric malpractice or ineffective practice? Please mention? ኣብ ምውላድ ዙርያ ብመረዳእታ ዘይተደገፉ/ብልመዲ ካብ ዝወሃቡ እንክብካቤታት ካብ ዝሰማዕክዮም ክትዝርዝርለይ ትክእሊ ዶ? | | | Mentioned/ **እወ** | Not mentioned  **ኣይፋሉን** |  | |
|  | 1. Use of enema**/** ሕፅቦ መዓንጣ | | | 1 | 0 |  | |
|  | 1. Perineal/Pubic shaving**/** ምልፃይ ፀጉሪ ብልዕቲ ተገልጋሊት | | | 1 | 0 |  | |
|  | 1. Apply fundal pressure to hasten delivery of baby or placenta/ ሕፃን ወይ መዳሕንቲ ተሎ ክዉለድ ተባሂሉ ኣብ ከብዲ ኣደ ሓይሊምፅቃጥ | | | 1 | 0 |  | |
|  | 1. Slap newborn/ ናፅላ ሕንጦ ምጥፍጣፍ/ መዓኮሩ ምጥፍጣፍ | | | 1 | 0 |  | |
|  | 1. Hold newborn upside down/ ናፅላ ህፃን ምሰተወለደ ብእግሩ ሒዝካ ንቑልቁል ኣፉ ምንጥልጣል | | | 1 | 0 |  | |
|  | 1. Excessive stretching of the perineum during the second stage of labor/ኣብ ካልኣይ ደረጃ ሕርሲ ንብልዕቲ ብክልቲኡ ኣፃብዕትኻ ገይርካ ክሰፍሕ ምግባር/ ምግታር | | | 1 | 0 |  | |
|  | 1. Restriction of oral fluids and food/ኣብ ወሊድ ግዘ ንወላድ ኣደ ምግብን ፈሳስን ከይትወስድ ምኽልካል | | | 1 | 0 |  | |
|  | 1. Something other than breast milk given to baby with in first hour of birth/ነቲ ህፃን ካብ ናይ ኣዶ ፀባ ወፃኢ ኣብ ውሽጢ ሓደ ስዓት ካሊእ ምግቢ ተወሂብዎ | | | 1 | 0 |  | |
| 218 | Do you allow your partner to enter to the delivery room? ሓጋዚ ወይ ብዓል ገዛኪ ናብቲ እትወልዱሉ ክፍሊ ክኣቱ ድልየት ኣለኪ ዶ? | | | | 1. No/ኣይፋሉን 2. Yes/እወ |  | |
| 219 | If yes Q218 why? /ንሕቶ ቁፅሪ 218 መልሰን እወ እንተኾይኑ፣ ንምንታይ? ____________________________________________________________________________________________________________________  _____________________________________________________________________________________________________________________ | | | | | | |
| 220 | If No for Q218, Why?/ ንሕቶ ቁፅሪ 218 መልሰን ኣይፋሉን እንተኾይኑ፣ንምንታይ? ______________________________________________ ____________________________________________________________________________________________________________________  _________________________________________________________________________________________________________ | | | | | | |
| 221 | Have you been involved in decision making for the type of care you have received in this facility?  ኣብዚ ጥዕና ትካል እዚ ዝረኸብክዮ ክንክንን ሕክምናን ኣብ ናይ ብዓልኪ ውሳነ ዝተመስረተ ድዩ ነይሩ? | | 1. No /ኣይፋሉን 2. Yes /እወ | | | |  |
| 222 | Have you faced any complication during this pregnancy/ኣብ እዋን ጥንሲኪ ዝኾነ ዓይነት ናይ ጥዕና ፀገም ኣጋጢሙኪ ነይሩ ድዩ? | | 1. No /ኣይፋሉን 2. Yes /እወ | | | |  |
| 223 | If yes for **Q222,** what type of complication do you face?  ንሕቶ ቁፅሪ 222 መልሰን እወ እንተኾይኑ፣ እንታይ ዓይነት ፀገም እዩ ኣጋጢሙኪ? | | 1. Hemorrhage/ ብርቱዕ መድመይቲ 2. PIH (Preeclampsia & Eclampsia) ልዑል ፀቕጢ ደም ኣብ ጥንሲ/ኢክላምፕሽያን ምንፍርፋርን/ 3. Infection / ረኽሲ 4. Other(specify)/ ካሊእ(ይገለፅ)_______________________ | | | |  |

**Part-III – Client Exit Checklist: Validating indicators of the quality of intrapartum and immediate post-partum care (ክፍሊ ሰለስተ፡ ዝወለደት ኣደ ካብ ትካል እንትወፅእ ብምሕታት ዝምላእ ናይ ፅሬት ኣገልግሎት መዐቀኒ ዝርዝር)**

| **S/No**  **(ተ.ቑ)** | **Task or Activity** (ስራሕቲ / ንጥፈታት) | **Correctly Performed**/ብትክክል ተሰሪሑ | | | | | | | **Skip to**  ናብ ቀፃሊ ሕቶ ይዘለል | |
| --- | --- | --- | --- | --- | --- | --- | --- | --- | --- | --- |
|  |  | Yes/እወ | No/ኣይሉን | | DK/ኣይፈልጦን | | | |  |  |
| 300 | Client code (መለለይ ቑፅሪ) _________________ ; MRN (ናይ ተገልጋሊት መለለይ ቑፅሪ) ____________________ | | | | | | | | | |
| 301 | Did the provider greet you and her companion (if present) respectfully (እቲ በዓል ሙያ ንዓኪ ወይ ምሳኪ ንዝመፀ ቤተሰብ ብዝግባእ ክብሪ ዝተማልአ ሰላምታ ሂቡኪ ዶ? | 1 | 0 | | 8 | | | |  | |
| 302 | Did the provider introduce you (at least tell one’s own name, profession/ role)/እቲ በዓል ሞያ ተፋሊጡኪ ዶ? (እንተናአሰ ሽሙ፣ ሞይኡ/ስራሕ ሓላፍነቱ ወዘተ… | 1 | 0 | | 8 | | | |  | |
| 303 | Did the provider actively listen you (able to respond for your concern, allocate adequate time to talk) (እቲ በዓል ሞያ ንሓሳብኪ ብፅሞና ኣዳሚፅዎዶ? | 1 | 0 | | 8 | | | |  | |
| 304 | Were you allowed to have a support person (companion) with you during your labor (ሓጋዚኺ /ባዓል ገዛኺ ኣብ መፅንሒ ክፍሊ ምሳኺ ንክህልይ ፈቒዶምልኪ ዶ? | 1 | 0 | | 8 | | | |  | |
| 305 | Did the health provider do rapid initial assessment (quick check of ABC)/ (እቲ በዓል ሞያ ናብ ጥዕና ትካል ኣብ እትኣትውሉ ቅልጡፍ ናይ መጀመርያ ዳህሳስ ጥዕና ገይርሉኪ ዶ? | 1 | 0 | | 8 | | | |  | |
| 306 | Did the health provider ask you about obstetric history (Name, Age, LMP, Gravidity, Parity and Past obstetric history) (እቲ ጥዕና በዓል ሞያ ኩነታት ታሪክ ጥንስኪ (እንተናኣሰ ሽምኪ፣ ዕድሜኪ፣ እትወልደሉ ግዘ፣ በዝሒ ጥንስኪ፣ በዝሒ ወሊድ፣ በዝሒ ቖልዑ፣ ዝሓለፈ ጥንስን ሕርስን ታሪክ) ሓቲቱኪ ዶ? | 1 | 0 | | 8 | | | |  | |
| 307 | Did the health provider ask you to obtainat least oral consent before examination and procedures/ እቲ በዓል ሞያ ቅድሚ ምርመራ ምግባሩን ሕክምናዊ ስራሕቲ /ፕሮሲጀር/ምፍፃሙን ፍቓድ ንክረክብ ሓቲቱኪዶ? | 1 | 0 | | 8 | | | |  | |
| 308 | During your time at this health facility for the birth of your baby, did someone ask you what your HIV status was? (ኣብዚ ትካል እዚ ኣብ እትወልደሉ ግዘ ኩነታት ኤችኣይቪ ምምርማርኪ ተጠይቕኪ ነይርኪ ዶ?) | 1 | 0 | | 8 | | | |  | |
| 309 | If not checked before, did someone offer youan HIV test (ንእንድሕር ዘይተመርሚርኪ ክትምርመሪ ተሓቲትኪ ዶ?) | 1 | 0 | | 8 | | | |  | |
| 310 | While you were at this facility for the birth of your baby, did any one test you for HIV? (ተመርሚራ ዶ?) | 1 | 0 | | 8 | | | |  | |
| 311 | Did the provider explainyou (support person) the procedures before proceeding (እቲ ዝወሃብ ኣገልግሎት በቢ ደረጅኡ ንባዕልኪ ወይ ንሓጋዚኪ ኣቐዲሙ ተነጊሩኪ ነይሩ ዶ? | 1 | 0 | | 8 | | | |  | |
| 312 | Did someone take your temperature (ሙቐት ተለኪዕኪ ነይርኪ ዶ?) | 1 | 0 | | 8 | | | |  | |
| 313 | Did someone take your pulse (ውቂዒት ልቢ ተለኪዓ ዶ?) | 1 | 0 | | 8 | | | |  | |
| 314 | Did someone take blood pressure (ፀቕቲ ደም ተለኪዕኪ ነይርኪ ዶ?) | 1 | 0 | | 8 | | | |  | |
| 315 | Did someone ask you to give them a urine sample, at or near your admission to the facility (ናብዚ ጥዕና ትካል እዚ ምስ ኣተኪ ሽንትምርመራተገይሩልኪ ነይሩ ዶ?) | 1 | 0 | | 8 | | | |  | |
| 316 | Did the provider conduct you abdominal examination –checks fetal heart beat with fetoscope /doppler /ultrasound (ኣካላዊ ናይ ከብዲ ምርመራተሰሪሑሉኪዶ፤ናይ ህፃንኪ ትርግታ ልቢ እውን?) | 1 | 0 | | 8 | | | |  | |
| 317 | Did the provider washes his/her hands with soap and water or uses antiseptic before examining you / በዓል ሞያ ቅድመን ድሕሪን ዝኾነ ዓይነት ምርመራ ሕክምና ምግባሩ ኢዱ ተሓፂቡ ዶ? | 1 | 0 | | 8 | | | |  | |
| 318 | Did the provider wear rubber gloves during vaginal examinations?  በዓል ሞያ ቅድሚ ብልዕታዊ ምርመራ ምግባሩ፣ ዝተዓሸገ ጓንቲ ይጥቀም ዶ ነይሩ? | 1 | 0 | | 8 | | | |  | |
| 319 | Did the provider clean your vulva with antiseptic solution during vaginal examinations  ቅድሚ ብልዕታዊ ምርመራ ምግባሩ፣ ደጋዊ ብልዕቲኪ ብፀረ ረኽሲ ፈሳሲ ፀሪጉዎ ዶ ነይሩ? | 1 | 0 | | 8 | | | |  | |
| 320 | Did the provider perform vaginal examination (ብልዕታዊ ምርመራ ገይሩልኪ ዶ?) | 1 | 0 | | 8 | | | |  | |
| 321 | Were you allowed to get up and walk or ambulate around while you were in labor? (እቶም ሰብ ሙያ ጥዕና ኣብ ቀዳማይ ደረጃ ሕርሲ እናሃለኪ ንክትንቀሳቐሲ ይፈቅዱልኪ ዶ ነይሮም? | 1 | 0 | | 8 | | | |  | |
| 322 | Did a health care provider ask you what position you wanted to choose during your labor OR  For the delivery of your baby?(ሰብ ሞያ ጥዕና ኣብ ግዘ ወሊድኪ ብዝደለክዮኣንፈት ኣደቓቕሳ (ፓዝሽን) ንኽትድቅሲየበራታትዑኩን ይፈቅድልኩን ዶ ኔሮም? | 1 | 0 | | 8 | | | |  | |
| 323 | Were you allowed to drink liquids or eat any food while you were in labor (እቶም ሰብ ሙያ ጥዕና ኣብ ቀዳማይ ደረጃ ሕርሲ እናሃለኪ ፈሳሲ ንክትሰትይ ወይ ድማ ምግቢ ንክትበልዒ የበራታትዑኪን ይፈቅዱልክን ዶ ነይሮም? | 1 | 0 | | 8 | | | |  | |
| 324 | Did you have a companion with you during delivery? / ከሕርሰኪ ሓቢሩኪ ንዝመፀ ብዓል ገዝኪ ወይ እውን ካሊእ ሰብ ናብ መዋለዲ ክፍሊ ክኣቱ ፈቒደምሉ ዶ ነይሮም? | 1 | 0 | | 8 | | | |  | |
| 325 | Did the provider follow your labour progress using Parthograph? ሰብ ሙያ ጥዕና ከይዲ ምውላድኪ ፓርቶግራፍ ብዘይምቁራፅን ብትኽክልን ተከታቲሎምኪ ዶ? | 1 | 0 | | 8 | | | |  | |
| 326 | Did the provider clean the delivery surface during your delivery (እቲ ብዓል ሞያነቲ እትዋለድሉ ኣልጋ /መዋለዲ ኣፅሩይዎዶ ነይሩ? | 1 | 0 | | 8 | | | |  | |
| 327 | Just after the delivery of your baby in the first few minutes after the delivery of your baby did any one give you a correct Active Management of Third Stage of Labor (AMTSL)/ ህፃንኪ ድሕሪ ምውላድ ርእሱ ቅልጠፍን ፅፉፍን ኣታኣላልያ ሳልሳይ ደረጃ ሕርሲ ብዝምልከት፡   1. Correctly administer uterotonic (timing, doseand route)/ማህፀን መኮምተሪ ዝበሃልመድሓኒት ብትኽክል (ኣብ ሰዓቱን፣ ቦትኡን መጠኑ ብዝሓለወን መንገዲ) ተዋሂቡኪ ዶ? | 1 | 0 | | 8 | | | |  | |
|  | 1. Provide controlled cord traction (CCT) / ዕትብቲ ቀስ ኣቢሉ ብምስሓብ ንኽወፅእ ሓገዝ ገይሩልኪ ዶ? | 1 | 0 | | 8 | | | |  | |
|  | 1. Firmly massage your lower abdomen to help make your womb contract (become firm)/ መዳሕንቲ ምስወፀ መድመይቲ ከየጋጥሙኪ ማህፀን ብምድራዝ ሓገዝ ገይሩልኪ ዶ? | 1 | 0 | | 8 | | | |  | |
| 328 | Did the provider examine your placenta and membranes for completeness and abnormalities/ እቲ መዳሕንቲ፣ ዝተመጮቐ ከይህልዎ፣ ኩሉ ተወሊዱ ምኳኑን ዘይንቡር ኣፈጣጥራ ከይነብሮን ግቡእ ምርመራ ብምግባር ኣረጋጊፁልኪ ዶ? | 1 | 0 | | 8 | | | |  | |
| 329 | In your first physical examination/check after delivery, did a health provider do a perineal and vaginal lacerations exam/ድሕሪ ምውላድኪ ምቕዳድ ብልዕትን ፍንዶትን ከይህልወኪ እቲ ብዓል ሞያ መርሚሩኪ ነይሩ ዶ? | 1 | 0 | | 8 | | | |  | |
| 330 | Did the provider make a follow-up, monitoring of your vital signs and amount of external blood loss immediately after delivery/ ድሕሪ ምውላድኪ መድመይቲ ከየጋጥሙክን፣ መሰረታዊ ኩነታት ጥዕናኪ ጥቡቕ ክትትል/ ፀቕጢ ደም፣ ዉቅዒት ልቢ/ ገይሩልኪ ዶ?   1. Check for bleeding (ምርግጋፅ ድሕሪ ወሊድ መድመይቲ) | 1 | 0 | | 8 | | | |  | |
|  | 1. Take BP (ምልካዕ ፀቕጢ ደም) | 1 | 0 | | 8 | | | |  |  |
|  | 1. Check for Perinal and vaginal lacerations (ምስንጣቕ ብልዕቲ ወይ እዉን ፈንዶት) | 1 | 0 | | 8 | | | |  |  |
|  | 1. Take temperature (ምልካዕ ሙቐት ኣዶ) | 1 | 0 | | 8 | | | |  |  |
|  | 1. Does the Woman ask for any pain relief medication during labor, delivery or immediate postpartum? (ኣብ እዋን ወሊድቃንዛ መዐገሲ መድሓኒት ንምርካብ ተሓቲትኪ ዶ?) | 1 | 0 | | 8 | | | |  |  |
|  | 1. Was the woman given any pain relief medication during labor, delivery or immediate postpartum? (ኣብ እዋን ወሊድ ቃንዛ መዐገሲ መድሓኒት ተዋሂቡኪ ዶ?) | 1 | 0 | | 8 | | | |  |  |
| 331 | Components of essential new-born care, Was your provider፡   1. Properly dried off and wrapped of your new-born /ነቲ ናፅላ ህፃንኪ ምሰተወለደ ብግቡእ ፀሪጉ ራሕሲ ንክደርቕ ተሸፊኑ ተቀሚጡ ዶ? |  | 0 | | 8 | | | |  | |
|  | 1. Ties or clamps and cut cord after birth / ዕትብቲናፅላ ህፃንኪብግቡእ ብምእሳር ፅሬቱ ብዝሓለወ መሳርሒ ዶ ቆሪፀምሉ? | 1 | 0 | | 8 | | | |  | |
|  | 1. Place the baby on your chest(skin-to-skin) immediately after delivery   እቲ ናፅላ ምስተወለደ ሽዑ ንሽዑ ናብ ሑቕፊ ኣዲኡ ዶ ገይርዎ፣ ማለት ሙቐት ንኽረክብ ሰዉነት ንሰዉነት ዶ ክሑቖፍ ተገይሩ? | 1 | 0 | | 8 | | | |  | |
|  | 1. Assess Apgar score within 1st and 5th minutes/ኣብ ናይ መጀመርያን ሓምሻይን ደቓይቕ ኩነታት ምንቅስቓስ፣ትግሃት፣ ግብረ መልሲ፣ኣተነፋፍሳ ህፃንኪ ዶ ተለኪዑ? | 1 | 0 | | 8 | | | |  | |
|  | 1. Did you breast feed your baby with in the first hour after delivery? ህፃንኪ ኣብ ዉሽጢ ሓደ ሰዓት ጡብ ምጥባዉ ጀሚርኪሉ ዶ? | 1 | 0 | | 8 | | | |  | |
|  | 1. Apply eye care using antimicrobial drop or TTC ointment? ነቲ ዝተወልደ ናፅላኺ ናብ ዓይኑ ፀረ ባክተርያ(ቴትራሳይክልን) ሂቦምዎ ዶ? | 1 | 0 | | 8 | | | |  | |
|  | 1. Give Vitamin K, 1 mg IM/ ንናፅላ ህፃን ቫይታሚን K ብጭዋዳ እግሩ ተዋሂቡ ዶ? | 1 | 0 | | 8 | | | |  | |
|  | 1. Dry cord care or use of Chlorehexidine/ ዕትብቲ ናይቲ ዕሸል ክለሮሄክሲደን ብዝብሃል ፈሳሲ ተቐቢኡ ዶ? | 1 | 0 | | 8 | | | |  | |
| 332 | Does the provider record/register the activity/care she/he has done? ነቲ ህፃን ዝተገበረ ክንክን ጥዕና ኩሉ ብግቡእ ተመዝጊቡ ዶ? | 1 | 0 | | 8 | | | |  | |
| 333 | Did the provider maintain privacy in providing clinical care (keeps your audio privacy, visual privacy; use of partitions, cover etc…)/ እቲ በዓል ሞያ ሕክምናዊ ክንክን ኣብ ዝህበሉ ግዘ ዉልቃዊ ክብርኪ (Privacy) ሓልይልኪ ዶ? ንኣብነት: ሰዉነትኪ ብምሽፋን፣ ናይ ምክክር ድምፆም ከይስማዕ፡ ፎቶን ቪድዮን ካብምልዓን ምቕራፅን ወዘተ ሓሊዩ ዶ? | 1 | 0 | | 8 | | | |  | |
| 334 | The provider did not verbally abuse you/ እቲ በዓል ሞያ ከይተፃረፈ ግልጋሎት ዶ ሂቡኪ? | 1 | 0 | | 8 | | | |  | |
| 335 | Did the provider treat you equally without discrimination (Based on specific client attributes: race, ethnicity, age, language, HIV status, economic status & educational level, etc.)**/** እቲ በዓል ሞያ ብዘይ ምንም ኣፈላላይ/ ብማዕረ ግልጋሎት ሂቡኪ ዶ? (ማለት ብዘርኢ፣ ሃይማኖት፣ ብቛንቛ፣ ብኩነታት ሃፍቲ፣ ት/ቲ ደረጃን ኩነታት ሕማም ኤች ኣይቪን ኣፈላላይ እንተይገበረ) | 1 | 0 | | 8 | | | |  | |
| 336 | Did the provider responds professionally when you ask for help/ እቲ በዓል ሞያ ንዝሓተትክዮ ሕቶ ሞያዊ ስነምግባር ብዝጠልቦ መንገድን፣ ደንቢን ስርዓትን መሊሱል ኪ ዶ? | 1 | 0 | | 8 | | | |  | |
| 337 | Did the provider give you adequate information regarding your treatment and care (about the problem, prognosis, & discharge criteria etc…)/ እቲ በዓል ሞያ ብዛዕባ እቲ ዝወሃበኪ መድሓኒትን ዝግበር ክንክን ጥዕናን እኹል ሓበሬታን ረኪብኪ ዶ? | 1 | 0 | | 8 | | | |  | |
| 338 | The provider did not physically abuse you during birth (slapping, pinching etc…)/ እቲ በዓል ሞያ ኣካላዊ ማህሰይቲ ከም ምውቓዕ፡ምቁርጣጥን ከየብፀሐ ግልጋሎት ሂቡኪ ዶ? | 1 | 0 | | 8 | | | |  | |
| 339 | The provider did not abandon you without care /በቲ በዓል ሞያ ሕክምናዊ ኣገልግሎት ከይረኸብኪ ንነዊሕ ግዜ ተኸልኪልኪ ፀኒሕኺ ዲኪ? | 1 | 0 | | 8 | | | |  | |
| 340 | Did the provider have good communication and collaboration with you, and colleague/እቲ በዓል ሞያ ምሳኽን ምስ መሳርሕቱን ፅቡቐ ርክብን ምትሕግጋዝን ነይርዎ ዶ? | 1 | 0 | | 8 | | | |  | |
| 341 | Does the facility ensures safe and clean care environment for clients (ensuring both coach, and equipment are clean)/እቲ ጥዕና ትካል ንተሓከምቱ ዉሑሱን ፅሩይ ከባብያዊ ግልጋሎትን ይህብ ድዩ? (መዋለዲ ኣልጋ፣ እንጥቐመሎም ኣቑሑን ካልኦትን)፡፡ | 1 | 0 | | 8 | | | |  | |
| 342 | Did the provider gave you appropriate counseling and Health education at time of discharge (Care provision) on the following components:  እቲ ብዓል ሙያ ካብቲ ትካል ጥዕና ቅድሚ ምዉፃእኪ ኣብዞም ኣብ ታሕቲ ተዘርዚሮም ዘለዉ ነጥብታት መሰረት ብምግባር ግቡእ ኣስተምህሮን ምኽርን ሂቡኪ ዶ?   1. Perinal care (Physiology of lochia, episiotomy care if there is present etc…) ኣተሓሕዛ ፅሬት ፈንዶት ባህርያት ፈሳሲ ድሕረ ወሊድ፣ ክንክን ምቕዳድ መትደብ ብልዕቲ እንተሃልዩ | 1 | 0 | | 8 | | | |  | |
|  | 1. Exclusive breast feeding & breast care/ ብዛዕባ ምጥባዉ ፀባ ጡብ ኣደ ጥራሕን ክንክን ጡብን | 1 | 0 | | 8 | | | |  | |
|  | 1. Family Planning- birth spacing/ትልሚ ስድራን፡ ኣረሓሒቅካ ምዉላድ ብዝምልከት | 1 | 0 | | 8 | | | |  | |
|  | 1. Immunization and other prophylaxis/ ክትባትን ፀረ ካልኦት ቅድመ ሕማማት ምክታብን | 1 | 0 | | 8 | | | |  | |
|  | 1. Self-care & other healthy behaviors/ ዉልቃዊ ፅሬትን ካልኦት ባህርያት ጥዕናን ብዝምልከት | 1 | 0 | | 8 | | | |  | |
|  | 1. Nutrition support/ ስነ መኣዛዊ ሓገዛት | 1 | 0 | | 8 | | | |  | |
|  | 1. Counsel delayed bathing of the new-born till 24 hrs/ሕንጦ ድሕሪ 24 ሰዓት ሰዉነቱ ክሕፀብ ከምዘለዎ ኣስተምህሮ ምሃብ | 1 | 0 | | 8 | | | |  | |
|  | 1. Review possible complication and readiness plan, both Newborns and mother danger sign before discharge/ ካብ ትካል ቅድሚ ምዉፃእኪ፣ከጋጥሙኪ ንዝኽእሉ ሓልክታት ጥዕናን ከመይ ክትምክትዮም ከም ዘለክን፣ ሓደገኛ ምልክታት ዝበሃሉን ኣመልኪቱ ትልሚ ንኽትገብሪ ፈተሸ ተገይሩልኪ ዶ? | 1 | 0 | | 8 | | | |  | |
|  | 1. Schedule the next PNC visit and thank the woman and her family member for coming ንዝቕፅል ድሕረ ወሊድ ክትትል መዓዝ ክትመፅኢ ከምዘለክን ቆፀሮ ምሃብን፣ ንባዕልክን ንቤተሰብክን ናብ ትካልና መፂእኪ ግልጋሎት ስለዝረኸብኪ ምስጋና ሂቡኪ ዶ? | 1 | 0 | | 8 | | | |  | |
| 343 | Did you checked and discharged by senior staff of the facility (the most experienced staff)/ ናብ ገዛኺ ቅድሚ ምምላስኪ በቲ ዝለዐለ ልምዲ ዘለዎ በዓል ሞያ ኩነታት ጥዕናኪ ክፃረ ተገይሩ ድዩ ትወፂ ዘለኪ? | 1 | 0 | | 8 | | | |  | |
| 344 | Did the provider practice any of the following activities during your childbirth and immediate postpartum care process? (CIRCLE ALL THAT APPLY)እቲ በዓል ሞያ ካብቶም ኣብ ታሕቲ ተዘርዚሮም ዘለዉ ተግባራት ተዋሂቦምኪ ዶ ነይሮም;   1. Use of enema/ ሕፅቦ መዓንጣ | Yes  1 | No  0 | | D/K  8 | | | |  | |
|  | 1. Perinal/Pubic shaving/ ምልፃይ ፀጉሪ ብልዕቲ ተገልጋሊት | 1 | 0 | | 8 | | | |  | |
|  | 1. Apply fundal pressure to hasten delivery of baby or placenta ሕፃን ወይ መዳሕንቲ ተሎ ክዉለድ ተባሂሉ ኣብ ከብዲ ኣደ ብምፅቃጥ ሓይሊ ምሃብ | 1 | 0 | | 8 | | | |  | |
|  | 1. Slap newborn/ ናፅላ ሕንጦ ምጥፍጣፍ/ መዓኮሩ ምጥፍጣፍ | 1 | 0 | | 8 | | | |  | |
|  | 1. Hold newborn upside down/ናፅላ ህፃን ምሰተወለደ ብእግሩ ሒዝካ ንቑልቁል ኣፉ ምንጥልጣል | 1 | 0 | | 8 | | | |  | |
|  | 1. Excessive stretching of the perineum during the second stage of labor   ኣብ ካልኣይ ደረጃ ሕርሲ ንብልዕትኪ ብክልቲኡ ኣፃብዕቱ ገይሩ ክሰፍሕ ምግባር/ ምግታር | 1 | 0 | | 8 | | | |  | |
|  | 1. Routine aminotomy /ኮነ ኢልካ ኩሉ ግዘ ቀሰተነሽተ ንክፈስስ ምግባር | 1 | 0 | | 8 | | | |  | |
|  | 1. Restriction of oral fluids and food/ኣብ ወሊድ ግዘኪ ምግብን ፈሳስን ከይትወስዲ ተኸልኪልኪ ነይርኪ ዶ? | 1 | 0 | | 8 | | | |  | |
|  | 1. Digital vaginal examination less than four hours ኣርባዕተ ሰዓት እንተይመልአ ተሎ ተሎ ብልዕታዊ ምርመራ ይካየደልኪ ዶ ነይሩ | 1 | 0 | | 8 | | | |  | |
|  | 1. For your delivery, shortly before you delivered your baby, did any one cut the opening of your vagina (episiotomy) to make more room for the baby’s head?ናይቲ ህፃን ርእሲ ቶሎ ንምውላድ ምቅዳድ ብልዕቲ ብኣግባቡ ዶ ተሰርሒሉኪ? | 1 | 0 | | 8 | | | |  | |
|  | 1. Routine intravenous fluid infusion for all laboring women ናብ ጥዕና ትካል ምስ መፃኪ ኢንፊዩዥን ፈሳሲ ተዋሂይቡኪ ዶ? | 1 | 0 | | 8 | | | |  | |
|  | 1. Something other than breast milk given to baby with in first hour of birth ነቲ ህፃን ካብ ናይ ባዕልኪ ፀባ ወፃኢ ሂብክዮ ድኪ? | 1 | 0 | | 8 | | | |  |  |
| 345 | What is the gestational age of the current newborn? / እቲ ህፃን ኣብ ክንደይ ዕድሚኡ ተወሊዱ | _______________Wks. | | | | | | | | |
| 346 | Did the provider measure the birth weight of newborn? / እቲ ብዓል ሙያ ነቲ ዝተወለደ ህፃን ክብደቱ መዚኑዎ ዶ? | 1 | | 0 | | 8 | |  | | |
| 347 | Did the provider caliber the scale & properly measure the birth weight? እቲ ብዓል ሙያ ቕድሚ ምምዛኑ ነቲ መምዘኒ ዓይርዎ ዶ? | 1 | | 0 | | 8 | |  | | |
| 348 | Did the birth weight of the newborn recorded? /እቲ ክብደት ህፃን ተመዚኑ ተመዝጊቡ ዶ? | 1 | | 0 | | 8 | |  | | |
| 349 | If recorded, how much was it?Please even if they are twin write the birth weight in grams /እንድሕር ተመዝጊቡ ነይሩ ፣ ክብደቱ ክንደይ ግራም እዩ?  *(መንታ እንድሕር ኮይኖም እውን ናይ ክሊትኦም ይመዝገብ)* | ________________grams/ብግራም | | | | | | | | |
| 350 | If <2000gm baby, what care was given? And what happened? /እንድሕር እቲ ህፃን ትሕቲ ክልተ ሺሕ ግራም ኮይኑ፣ እንታይ ዓይነት እንክብካቤ ተዋህይብዎ፤ካብኡስ እንታይ ኣጋጢሙዎ ? | ______________________________________________ | | | | | | | | |
| 351 | If <2000gm, is the baby on KMC? እቲ ዝተወለደ ህፃን *እንድሕር* ትሕቲ ክልተ ሺሕ ግራም ኮይኑ ሕቑፈ ኣብ ጎጋ ተጀሚሩሉ ዶ? | 1. No/ኣይፋሉን 2. Yes/ እወ | | | | | | | | |
| 352 | If the baby is on KMC, did the provider record the progress in accordance with the checklist?  እቲ ዝተወለደ ህፃን ሕቑፈ ኣብ ጎጋ እንድሕር ተጀሚሩሉ፣እቶም ሰብ ሙያ ከይድን ለውጥን ጥዕና ናይቲ ህፃን በቲ መቖፃፀሪ ሓበሬታ መሰረት ዶ ይከታቶልዎ? | 1. No/ኣይፋሉን 2. Yes/ እወ | | | | | | | | |
| 353 | Did you experience any of the obstetric complications during or after your delivery?ነዚ ህፃን ክትወልዲእንከለኪ ኣብዚ ትካል ዘጋጠመኪ ፀገም/ሓልኪ ጥዕና ነይሩ ድዩ? | 1 | | 0 | | 8 | |  | | |
| 354 | If yes for **Q353,** at what stage of Labor & Delivery did the complication occur? መልሲ ንሕቶ ቁፅሪ 353 እወ እንተኾይኑ፣ ኣበየናይ ደረጃ ሕርሲ እዩ ክትወልዲ እንከለኪ አቲ ፀገም ዘጋጢሙኪ? | 1. During labour including admission/ሕርሲ ጀሚርኒ ናብ ትካል እንትኣትው ጀሚሩ 2. During delivery/ኣብ ግዘ ወሊድ 3. Postpartum(before discharge)   ድሕረ ወሊድ/ ኣብ ትካል እንከለኩ | | | | | | | | |
| 355 | If yes for **Q353**, can you tell me the type of complication you face?  መልስኪ ንቁፅሪ **353** እወ እንተኾይኑ ፣ ኣየናይ ዓይነት ፀገም ጥዕና እዩ ኣጋጢሙኪ? | 1. Hemorrhage/መደመይቲ 2. Preeclampsia & Eclampsia/ ቅድመምንፍርፋርን ምንፍርፋርን 3. Obstructed labour/ዝተዓገተ ሕርሲ 4. Sepsis/Infection/ረኽሲ 5. Tear/laceration/ምቐዳድ/ምልሓፅ ብልዕቲ 6. Maternal death/ሞት ኣደ 7. Other (specify)/ካሊእ/ይፀሓፍ/___________ | | | | | | | | |
| 356 | Did you experience any of the neonatal complication within the health facility before discharge? ዝኾነ ዓይነት ሓልኪ/ ፀገም ጥዕና ናፅላ ህፃን ኣጋጢሙኪ ነይሩ ድዩ? | 1 | | | | | 0 | | | 8 |
| 357 | If yes for **Q356,** what type of complication does newborn have? መልሰን ንቁፅሪ 356 እወ እንተኾይኑ ፣ ኣየናይ ዓይነት ፀገም ጥዕና እዩ እቲ ናፅላ ህፃን ዘጋጠሞ? | 1. Asphyxia/ምዕፋን/ ዘይምስትንፋስ 2. Still birth/ምዉት ምዉላድ 3. Infection/ረኽሲ 4. Early neonatal death within 24 hours/ ተይፀንሐ ኣብ ዉሽጢ 24 ሰዓት ሞት ናፅላ 5. Others (specify) ይገለፅ_________________ | | | | | | | | |
| 358 | Approximately how long from the time you delivered your baby did you stay in the facility? / ካብ ዝተጋላገልክሉ እስካብ ትወፅሉ/ ሐዚ/ ንክንደይ ሰዓት ፀኒሕኪ? | 1. Within 6 hours/ኣብ ዉሽጢ 6 ሰዓ 2. 6 to 12 hours / ኣብ 6 ክሳብ 12 ሰዓት ኣብዘሎ ግዘ ዉሽጢ 3. 12–24 hours/ካብ 12 ክሳብ 24 ሰዓታት ዉሽጢ 4. Greater than 24 hours/ድሕሪ 24 ሰዓታት | | | | | | | | |
| 359 | Were there any measure delays in needed treatment during your intrapartum and immediate postpartum care? ንክትወልዲ ናብ ትካል ካብ ዝኣተክሉ ሰዓት ጀሚሩ እስካብ ሐዚ ግልጋሎት ኣብ ምውሃብ ይዝንግዑኪ ነይሮም ዶ? | 1. No (ኣይፋሉን) 2. Yes (እወ) | | | | | | | | |

**የቕንየለይ !!**
